# Hypersensitivity associated with molar-incisor hypomineralisation (MIH) among elementary schoolchildren in Bavaria, Germany: results from a cross-sectional study

**DOI:** 10.1007/s40368-025-01054-1

**Published:** 2025-06-04

**Authors:** R. Gaballah, S. Amend, K.-F. Fresen, H. Schill, R. Michel, V. Pitchika, J. Kühnisch, N. Krämer

**Affiliations:** 1 University Medical Centre Giessen and Marburg (Campus Giessen), Medical Centre for Dentistry, Department of Paediatric Dentistry, Justus-Liebig-University Giessen, Giessen, Germany; 2 University Hospital, Department of Conservative Dentistry and Periodontology, Ludwig-Maximilians-University Munich, Munich, Germany; 3 Customer Analytics, Deutsche Bank, Frankfurt am Main, Germany

**Keywords:** Epidemiology, Dental enamel hypomineralization, Hypersensitivity, Molar hypomineralization, Post-eruptive breakdown

## Abstract

**Purpose:**

This cross-sectional epidemiological study aimed to provide population-based data on hypersensitivity associated with molar–incisor hypomineralisation (MIH) in 8- to 10-year-olds from Bavaria, Germany. It was hypothesized that hypersensitivity would be equally distributed among MIH teeth.

**Methods:**

A total of 5418 schoolchildren (8–10 years) were examined using the MIH criteria of the European Academy of Paediatric Dentistry (EAPD) and the MIH Treatment Need Index (MIH-TNI). MIH-TNI 1 was linked with mild MIH; MIH-TNI 2–4 corresponded to severe MIH. Hypersensitivity was recorded dichotomously (yes/no) after a two-second, 2.8-bar air blast (Schiff test). Descriptive statistics and a mixed-effects logistic regression model—adjusted for age, sex, region, tooth type, and caries status—explored hypersensitivity in MIH-affected teeth.

**Results:**

The MIH prevalence was 17.5% (*n* = 945). In this group, 9.8% of the children showed hypersensitivity in at least one tooth; 5.6% of all MIH-affected teeth were hypersensitive. Nearly half of the MIH-affected children (49.7%) presented severe MIH-TNI findings; MIH-TNI 2 was the most frequent finding (39.9%). Regression analyses indicated that demarcated opacities were significantly less likely to be associated with hypersensitivity (aOR = 0.054, *p* < 0.001). However, enamel breakdown did not show a significant association with hypersensitivity (aOR = 0.853, *p* = 0.693).

**Conclusion:**

Although MIH was relatively common, overall hypersensitivity rates were low. Demarcated opacities were significantly less prone to hypersensitivity, yet enamel breakdown did not significantly differ from healthy teeth. Further standardised epidemiological research is needed to clarify variations in hypersensitivity rates and explore additional risk factors, e.g., breakdown depth or defect extension.

## Introduction

Molar-incisor hypomineralisation (MIH) is a prevalent developmental disorder of enamel in children and adolescents that involves at least one affected first permanent molar and potentially the permanent incisors (Weerheijm et al. 2001). Clinically, MIH defects can be categorized into demarcated opacities, post-eruptive enamel breakdowns, atypical restorations, or extractions due to MIH. These criteria, introduced by the European Academy of Paediatric Dentistry (EAPD, Lygidakis et al. 2010, 2022), are well accepted and meanwhile widely used in MIH studies (Kühnisch and Fresen 2024). Individuals with MIH may experience discomfort while eating, drinking, or brushing their teeth, which are symptoms of reduced dental functionality and quality of life (Raposo et al. 2019; Vicioni-Marques et al. 2023).

In recent years, the hypersensitivity of MIH teeth has gained increasing attention scientifically. The hypersensitivity associated with MIH is caused primarily by the porous structure of hypomineralised enamel or enamel breakdowns, which fail to sufficiently protect the underlying dentin from thermal, mechanical, or functional influences (Ebel et al. 2018). Research by Rodd et al. (2007) suggested that increased expression of heat receptors, such as the transient receptor potential vanilloid 1 ion channel (TRPV1), in the pulp of hypomineralised teeth potentially explains hypersensitivity in MIH-affected teeth (Rodd et al. 2007). The severity of hypersensitivity can vary depending on the extent of enamel defects and dentin exposure. Recent studies have shown that MIH-affected teeth with post-eruptive breakdowns are more likely to exhibit hypersensitivity than teeth with only demarcated opacities (Raposo et al. 2019; Linner et al. 2021). However, the existing data remain controversial. For example, in the study by Raposo et al. (2019), a high prevalence of caries was reported alongside MIH, and dental caries was recognized as a confounding factor in hypersensitivity assessment.

To improve the documentation of hypersensitivity in MIH patients, the MIH Treatment Need Index (MIH-TNI) was developed, which cross-tabulates demarcated opacities and enamel breakdowns in relation to hypersensitivity (Steffen et al. 2017; Bekes et al. 2023). Clinical studies have revealed that tooth hypersensitivity in MIH children is common (Raposo et al. 2019; Linner et al. 2021; Vicioni-Marques et al. 2023). While the nature of MIH-related hypersensitivity has been described in several clinical (de Castro et al. 2021; Elhennawy et al. 2022; Joshi et al. 2022) and observational studies (Oliver et al. 2014; Petrou et al. 2014; Oyedele et al. 2015; Humphreys and Albadri 2020; Vicioni-Marques et al. 2023), only one epidemiological study has aimed to assess the prevalence of MIH-related hypersensitivity via a population-based approach. This recent study from Norway reported a prevalence rate of 25.8% among children with MIH (Afzal et al. 2024). However, this study considered hypersensitivity in the first permanent molars, excluding upper and lower central and lateral incisors; furthermore, the air blast test was not used consistently on all teeth, which potentially caused test bias.

When considering the clinical importance of MIH-related hypersensitivities in daily dental practice, surprisingly little information is available from the literature. In particular, the notable absence of epidemiological trials must be mentioned here. Therefore, this cross-sectional epidemiological study aimed to provide population-based data on MIH-related hypersensitivities in a paediatric population. It was hypothesized that hypersensitivity is evenly distributed across all MIH-affected teeth.

## Materials and methods

### Study design and participants

This cross-sectional epidemiological study was conducted from March to July 2023. It involved voluntary dental examinations of 5418 schoolchildren in the 3rd and 4th grades, aged between 8 and 10 years, across 87 elementary schools. The initiative was a collaborative effort between the state working group for public dental health (Landesarbeitsgemeinschaft Zahngesundheit e.V., LAGZ), Justus-Liebig-University (JLU) Giessen, and Ludwig-Maximilians-University (LMU) Munich. Ethical approval was obtained from the Ethics Committee of the Faculty of Medicine at JLU Giessen (AZ 72/22), adhering to the ethical guidelines of the 1964 Declaration of Helsinki and its later amendments.

### Sample size calculation and recruitment strategy

Following the recruitment strategy outlined by Fresen et al. (2024), the sampling approach was based on population distributions across Bavaria, with the aim of achieving a representative sample in terms of geography (with higher population densities in southern and central regions) and settlement structure (estimated 25% urban vs. 75% rural) (Bavarian Statistics Office). The minimum sample size of 5000 children was determined using an anticipated prevalence of 13.4% (Amend et al. 2021). Initially, 76 elementary schools were randomly selected and contacted for participation. Due to refusals and dropouts, additional schools were recruited in a second wave, resulting in a final sample of 87 participating schools. All 3rd and 4th grade children at these schools received study information and consent forms. In total, 10,749 children were invited to participate. Of these, 5418 children (50.4%) met the predefined inclusion criteria and were included in the present study.

### Clinical examination and diagnostic standards

Clinical examinations were performed under standardised conditions by four previously calibrated dentists, with the necessary equipment provided by the LAGZ. The inclusion criteria were a healthy ASA Class I health status (Mayhew et al. 2019), written parental consent, and the child’s assent to participate. The exclusion criteria included children with general illnesses classified under ASA Classes II–IV, lack of written parental consent, absence on the day of examination, or refusal by the child to participate.

Examinations took place in various school environments and were adapted to the local infrastructure. Depending on the available space, children were either seated on a portable examination table (PINO mobile massage bed “Atoll,” Pino Pharmazeutische Präparate, Hamburg, Germany) or on a regular classroom chair. Consistent illumination was ensured using mobile halogen lamps (Haeberle Halux 50S, 50 watt, Haeberle GmbH, Stuttgart, Germany) or high-intensity headlamps (Starlight nano1, starMed GmbH, Bad Oeynhausen, Germany). A standardised examination protocol was followed, using a mouth mirror (Marlin, product-no. 490R0, Carl Martin GmbH, Solingen, Germany) and a blunt Community Periodontal Index (CPI) probe (CP-11.5B6, Hu-Friedy, Chicago, IL, USA). Before inspection, all tooth surfaces were dried using compressed air from a mobile compressor unit (SNR MAC-A-002670, DTS Design, Mammendorf, Germany) to enhance visibility. In cases of poor oral hygiene, visible plaque was either removed with cotton rolls or the child was asked to brush their teeth prior to the examination.

**Fig. 1:**
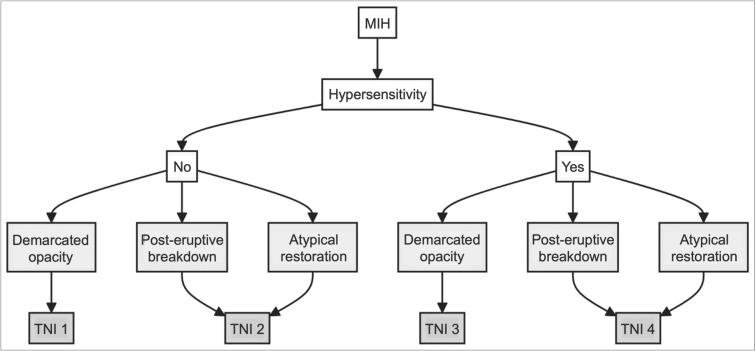
Classification of enamel hypomineralisation according to the MIH Treatment Need Index (MIH-TNI) (Bekes et al. 2023)

Enamel hypomineralisation in terms of MIH was assessed via the EAPD criteria (Lygidakis et al. 2010, 2022) on all first permanent molars and permanent incisors (upper and lower central/lateral incisors). According to the EAPD definition (Lygidakis et al. 2010, 2022), an MIH diagnosis requires at least one first permanent molar to exhibit demarcated opacities, post-eruptive enamel breakdowns, atypical restorations, or extractions due to MIH. Developmental defects were recorded only if the defect size exceeded 2 mm in diameter. Hypersensitivity was recorded at individual and tooth levels, with any subject presenting at least one hypersensitive MIH tooth being marked as having MIH hypersensitivity. For individuals or teeth without MIH, a negative observation was recorded. The evaluation was conducted by stimulating the occlusal surface of a tooth with a two-second air blast from a mobile dental air syringe at 2.8 bar pressure and room temperature. The air was applied perpendicularly from approximately 1 cm distance, with adjacent teeth shielded by the examiner’s fingers. The assessment used a binary method, observing the subject’s response to the air stimulus. If the subject did not react to the air stimulus or reacted without requesting its discontinuation, it was classified as not hypersensitive. Conversely, if the subject requested discontinuation of the stimulus, moved away from it, or found it painful, it was documented as hypersensitive (Schiff et al. 1994). In addition, the severity of MIH was classified via the MIH-TNI (Bekes et al. 2023). MIH-TNI classifications are defined as follows: TNI 1 identifies demarcated opacities without hypersensitivity, TNI 2 encompasses post-eruptive enamel breakdowns or atypical restorations in the absence of hypersensitivity, TNI 3 is assigned to opacities accompanied by hypersensitivity, and TNI 4 refers to post-eruptive enamel breakdowns or atypical restorations with hypersensitivity (Fig. 1). Cases classified under TNI 1 were considered mild, whereas severe cases were defined as TNI 2 or higher. The classification process was applied at both the individual tooth and patient levels, without further differentiation based on the extent of the defect (e.g., TNI 2/4 a, b, or c).

Additionally, the caries status of all permanent teeth was assessed independently of MIH, using the DMF/T index for cavitated lesions in accordance with the WHO protocol (World Health Organization 2013), and the ICDAS/UniViSS criteria for initial lesions (Kühnisch et al. 2009; Pitts 2009), as previously described in detail by Fresen et al. (2024).

### Examiner training and calibration

Prior to the study, four dentists underwent calibration through three-day training led by two experienced dentists and epidemiologists. This session covered the study design, indices, and diagnostic principles and included practical exercises with high-quality images of MIH lesions for discussion and differential diagnosis. Subsequently, 120 high-quality images depicting smooth and occlusal surfaces were assessed via the EAPD criteria (Lygidakis et al. 2010). Examiners independently evaluated these images, with a reanalysis conducted after approximately four weeks to ensure intra-rater reliability. Agreement levels were assessed via Cohen’s kappa (Cohen 1960), which is aligned with international guidelines. According to these guidelines, kappa values below 0 indicate poor agreement, 0–0.2 represent slight agreement, 0.21–0.4 fair, 0.41–0.6 moderate, 0.61–0.8 substantial, and 0.81–1.00 almost perfect agreement (Landis and Koch 1977). The registered kappa values for all examiners demonstrated substantial to almost perfect agreement among examiners, with intra-examiner reliability scores ranging from 0.484 to 1.0 and inter-examiner reliability ranging from 0.371 to 0.956.

### Data management and statistical analysis

Examination data were initially recorded on paper and then transferred into EpiData (version 4.6, EpiData Association, Odense, Denmark). Data management and preliminary analyses were conducted via Microsoft Excel (Microsoft Corporation, Redmond, WA, USA). All further statistical analyses were performed in SPSS version 29.0.1.0 (IBM, Armonk, NY, USA) and Stata/MP 18.0 (StataCorp, College Station, TX, USA). Descriptive statistics (frequencies, means, standard deviations) were used to summarize the sample. Additionally, a mixed-effects logistic regression model was employed to examine the association between MIH diagnosis and hypersensitivity at the tooth level. MIH diagnosis served as the exposure variable and was categorized into four groups: healthy, demarcated opacity, enamel breakdown and atypical restoration. The binary outcome variable was hypersensitivity (yes/no) on each tooth. To account for potential confounders, the model was adjusted for age, sex (female/male), region (urban/rural), type of tooth (anterior/posterior) and caries diagnosis in first permanent molars (healthy/initial caries/decayed/filled). Furthermore, to address the intraindividual clustering of teeth, patient ID was included as a random effect. The results were reported as adjusted odds ratios (aORs) with 95% confidence intervals (CIs) for each independent variable. A *p*-value < 0.05 was considered statistically significant.

## Results

**Fig. 2:**
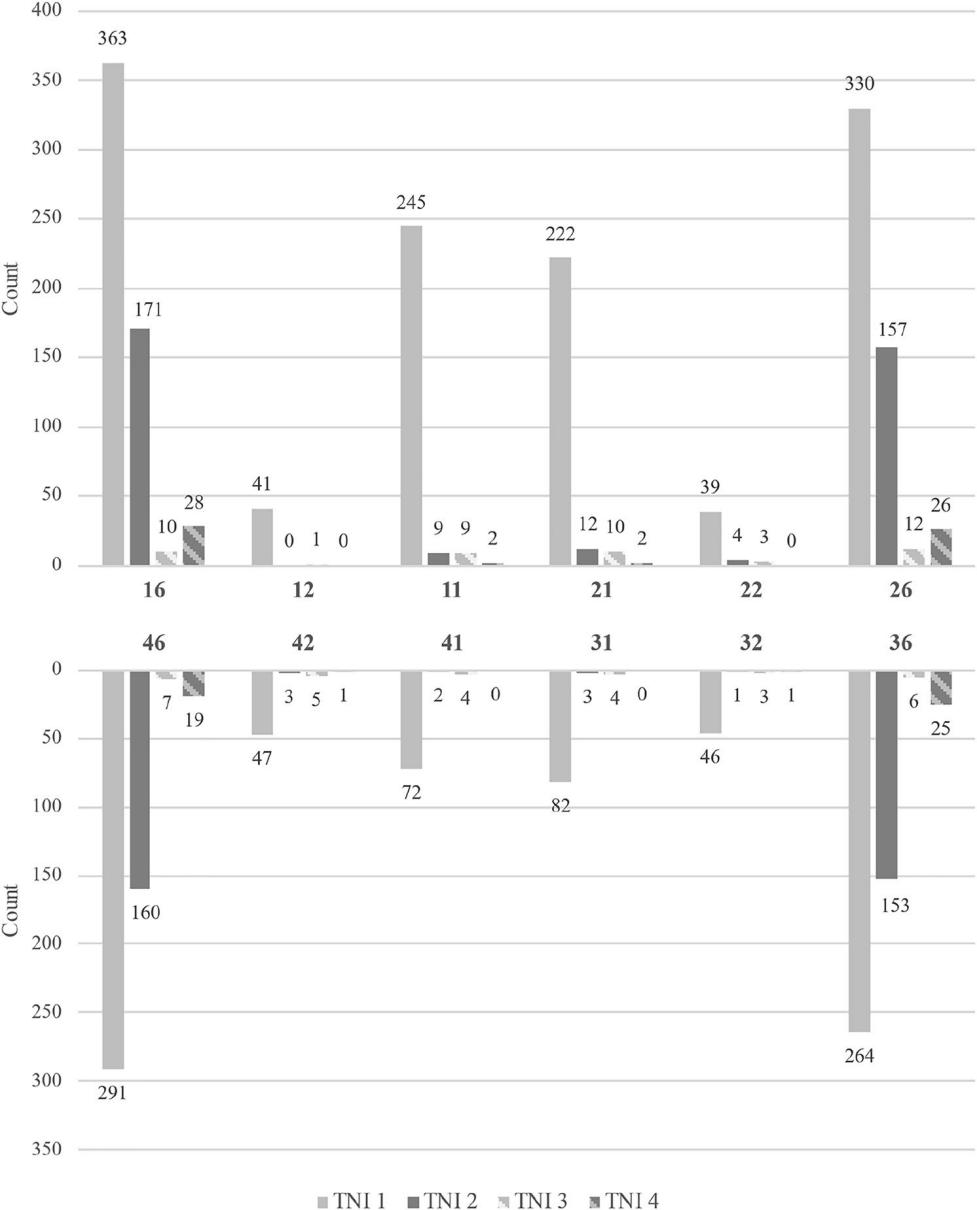
Tooth-related distribution of the categories of the MIH Treatment Need Index (MIH-TNI). A total of 2895 MIH-affected index teeth were documented across 945 individuals diagnosed with MIH

This cross-sectional epidemiological study included 5418 children aged 9.8 ± 0.7 years (2692 males and 2726 females). MIH was diagnosed in 945 children, accounting for 17.5% of the sample. One subject had only MIH-related extractions and was excluded from the analysis. Among the 65,016 index teeth examined (first permanent molars and upper/lower central and lateral incisors), 4.5% (*n* = 2895) exhibited MIH (Fig. 2). A total of 3290 MIH-affected teeth, including additional affected teeth beyond these index teeth, were documented. Within the MIH-affected group, hypersensitivity was noted in 9.8% (*n* = 93) of the children and 5.6% (*n* = 185) of all the MIH-affected teeth (Table 1).

**Table 1:** Frequency of hypersensitivities in relation to the diagnostic criteria of MIH at the individual and tooth levels in 8- to 10-year-olds from Bavaria, Germany

Variable	Hypersensitivity				Total	
	No		Yes			
	*N*	%	*N*	%	*N*	%
Individual level (*n* = 945)						
Children with demarcated opacities only	475	50.3	21	2.2	496	52.5
Children with at least one post-eruptive enamel breakdown	366	38.7	71	7.5	437	46.2
Children with at least one atypical restoration	11	1.2	1	0.1	12	1.3
Total	852	90.2	93	9.8	945	100.0
Tooth level (*n* = 3290)						
Permanent teeth with demarcated opacities only	2427	73.8	84	2.5	2511	73.1
Permanent teeth with post-eruptive enamel breakdown	404	12.3	61	1.9	465	16.1
Permanent teeth with atypical restoration	274	8.3	40	1.2	314	10.8
Total	3105	94.4	185	5.6	3290	100.0

Individual level refers to the most severe MIH condition observed in each child. From better to worse: demarcated opacities < atypical restoration < post-eruptive enamel breakdown. No hypersensitivity < hypersensitivity

When comparing clinical presentations, hypersensitivity was observed less frequently (2.2% of children and 2.5% of teeth) in cases with only demarcated opacities than in those with post-eruptive enamel breakdowns (7.5% of children and 1.9% of teeth). Hypersensitivity associated with atypical restorations was the least common, affecting 0.1% of children and 1.2% of teeth. Overall, the first permanent molars were most frequently associated with hypersensitivity, whereas permanent incisors were affected considerably less often (Fig. 2).

The distribution of cases according to the MIH-TNI index is shown in Table 2. Among all individuals with MIH, 50.3% (*n* = 475) had only demarcated opacities without hypersensitivity (MIH-TNI 1), whereas 49.7% (*n* = 470) presented signs of more severe hypomineralisation. Specifically, post-eruptive enamel breakdown without hypersensitivity (MIH-TNI 2: *n* = 377; 39.9%) was more common than enamel breakdown accompanied by hypersensitivity (MIH-TNI 4: *n* = 72; 7.6%) or demarcated opacities with hypersensitivity (MIH-TNI 3: *n* = 21; 2.2%). When the reasons for severity were considered, cases with hypersensitivity were less common than those with enamel breakdown. Analysis of tooth-related data indicated that mild findings (MIH-TNI 1) were registered in 73.8% of all MIH-affected teeth, whereas 26.2% exhibited signs of more severe diagnoses. Once again, enamel breakdowns were more common than hypersensitivity (Table 2).

**Table 2:** Case distribution among children and teeth diagnosed with MIH in relation to the categories of the MIH Treatment Need Index (MIH-TNI)

Variable	Hypersensitivity			
	No		Yes	
	*N*	%	*N*	%
Individual level (*n* = 945)				
Children with demarcated opacities only	TNI 1		TNI 3	
	475	50.3	21	2.2
Children with at least one post-eruptive enamel breakdown/atypical restoration	TNI 2		TNI 4	
	377	39.9	72	7.6
Tooth level (*n* = 3290)				
Permanent teeth with demarcated opacities only	TNI 1		TNI 3	
	2427	73.8	84	2.5
Permanent teeth with post-eruptive enamel breakdown/atypical restoration	TNI 2		TNI 4	
	678	20.6	101	3.1

*TNI 1* demarcated opacities without hypersensitivity, *TNI 2* post-eruptive enamel breakdown or atypical restoration without hypersensitivity, *TNI 3* demarcated opacities with hypersensitivity, *TNI 4* post-eruptive enamel breakdown or atypical restoration with hypersensitivity

**Fig. 3:**
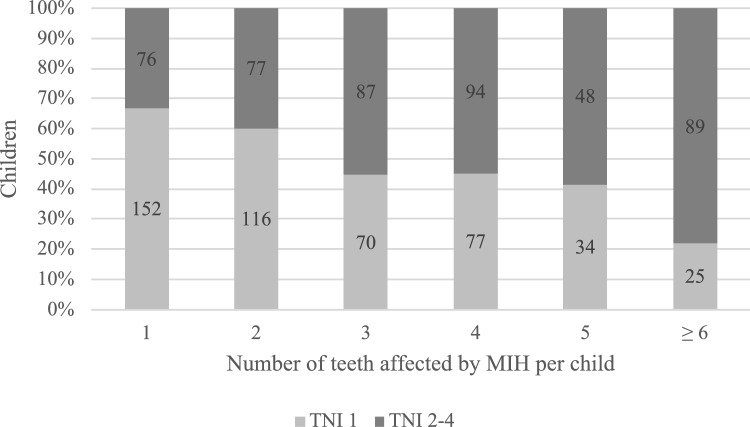
Distribution of MIH severity (TNI 1 vs. TNI 2–4) in relation to the number of teeth affected by MIH per child. The proportion of children with severe MIH (TNI 2–4) increases with the number of affected teeth

A mixed-effects logistic regression model was fitted to determine whether hypersensitivity was associated with the type of MIH diagnosis and other covariates (Table 3). The presence and condition of MIH were found to influence the occurrence of hypersensitivity. Specifically, demarcated opacities were significantly less likely to be associated with hypersensitivity than healthy teeth (aOR = 0.054, *p* < 0.001). However, enamel breakdown did not show a significant association with hypersensitivity (aOR = 0.853, *p* = 0.693). Sex also had a significant effect on hypersensitivity in the model; males (aOR = 0.389, *p* = 0.041) exhibited fewer hypersensitive teeth than females did (Table 3). However, age, region, tooth type and caries diagnosis in the first permanent molars did not significantly affect hypersensitivity. Additionally, with an increasing number of MIH-affected teeth per child, the severity of MIH findings tended to increase as well (Fig. 3). For children with only 1–2 MIH-affected teeth, the majority had an MIH-TNI of 1. In contrast, among children with 3 or more MIH-affected teeth, more severe forms became predominant. Statistical analysis revealed a significant association between MIH severity and the number of affected teeth per child (Pearson’s correlation = 0.270, *p* < 0.001).

**Table 3:** Distribution of MIH diagnosis on the tooth level and covariates; adjusted odds ratios (aORs) and their corresponding 95% confidence intervals from the mixed-effects logistic regression model for hypersensitivity in relation to MIH diagnosis, adjusted for age, sex, region, type of tooth and caries diagnosis in first permanent molars

Variable	Category	Case distribution				Mixed-effects logistic regression model	
		No hypersensitivity		Hypersensitivity		Hypersensitive versus non-hypersensitive teeth	
						aOR (95% CI)	*p* value
Age	Mean ± standard deviation	9.3 ± 0.7		9.3 ± 0.7		0.714 (0.400–1.275)	0.255
Sex	Female (*N*/%)	32,587	50.12	125	0.19	1 (Reference)	–
	Male (*N*/%)	32,241	49.59	63	0.10	0.389 (0.158–0.962)	0.041
Region	Urban (*N*/%)	20,055	30.85	57	0.09	1 (Reference)	–
	Rural (*N*/%)	44,773	68.86	131	0.20	0.992 (0.398–2.472)	0.986
Type of tooth	Anterior	43,289	66.58	55	0.08	1 (Reference)	–
	Posterior	21,539	33.13	133	0.20	0.660 (0.339–1.286)	0.222
MIH diagnosis	Healthy (*N*/%)	61,705	94.93	0	0.00	1 (Reference)	–
	Demarcated opacity (*N*/%)	2427	3.73	84	0.13	0.054 (0.022–0.132)	<0.001
	Enamel breakdown (*N*/%)	404	0.62	61	0.10	0.853 (0.388–1.874)	0.693
	Atypical restoration (*N*/%)	274	0.42	40	0.06	1 (–)	–
Caries diagnosis in first permanent molars	Healthy (*N*/%)	52,890	81.35	162	0.25	1 (Reference)	–
	Initial caries (*N*/%)	6224	9.57	16	0.02	0.667 (0.089–4.977)	0.693
	Decayed (*N*/%)	1404	2.16	0	0.00	1 (–)	–
	Filling (*N*/%)	4310	6.63	10	0.02	0.566 (0.087–3.689)	0.552

To adjust the multilevel nature of the dataset, the patient ID was included as a random effect

## Discussion

This cross-sectional epidemiological study recorded MIH and related hypersensitivity in 8- to 10-year-old Bavarian elementary schoolchildren. The results indicated an MIH prevalence of 17.5%, which is slightly higher than the global prevalence rates reported in the literature (Schwendicke et al. 2018; Lopes et al. 2021). Furthermore, the frequency of hypersensitivity at the population level can be considered low; only 9.8% of all 8- to 10-year-olds with MIH presented at least one hypersensitive tooth. At the tooth level, only 5.6% of all permanent teeth affected by MIH were hypersensitive, and the majority of these teeth presented post-eruptive enamel breakdown. Consequently, the initially formulated hypothesis must be rejected, as no equal distribution was found among the diagnostic criteria for MIH.

Overall, it must be emphasized that the prevalence of hypersensitivity among MIH-affected individuals in this study was lower than that reported in other studies on a global scale. However, drawing direct comparisons is challenging due to the considerable methodological differences and potential shortcomings of other studies. In clinical studies (Elhennawy et al. 2022; Joshi et al. 2022), hypersensitivity rates ranging from 25.8% to 56.6% have been reported, whereas observational studies (Oyedele et al. 2015; Petrou et al. 2015; Dantas-Neta et al. 2016; Menoncin et al. 2019) have reported proportions ranging from 15.0% to 98.3%. These discrepancies may, in part, be attributed to biases inherent in clinical or observational studies, which potentially oversample diseased individuals. In contrast, epidemiological studies can provide more reliable insights into population-based distributions owing to broader and less selective settings. The data presented here (Table 1) are, therefore, novel in this context, particularly compared with the only other epidemiological study on MIH-related hypersensitivity conducted in public dental clinics in Oslo, Norway (Afzal et al. 2024). In their cohort, a higher MIH prevalence (28.2%) and a higher hypersensitivity rate (25.8%) were reported. This discrepancy cannot be easily explained, as both studies applied a comparable epidemiological approach and used an air stimulus for clinical hypersensitivity assessment. However, key methodological differences persist. In the present study, hypersensitivity was recorded exclusively if a child explicitly requested discontinuation of the air stimulus, moved away from it, or perceived it as painful, thereby minimizing mild or ambiguous responses (Schiff et al. 1994). By contrast, Afzal et al. (2024) first asked each child whether they had experienced pain or hypersensitivity in the first permanent molars; if the answer was “yes,” the tooth was immediately registered as hypersensitive. Only if the child answered “no”, an air stimulus was applied to confirm the absence of hypersensitivity. Such subjective self-reporting in younger children may lead to overestimation, thus partially explaining the higher hypersensitivity rate in the Oslo cohort. Another noteworthy point is the difference in age: the children in Afzal et al. (2024) were on average 8.3 years old–about one year younger than in the present study. Consequently, their first permanent molars might have been erupted for a shorter period, increasing the likelihood that they were still untreated or more prone to sensitivity issues. In contrast, the third- and fourth-grade participants in our investigation typically had their first permanent molars erupted for two or more years, making it less likely for these teeth to remain symptomatic, as physiological processes, such as ongoing or reactive dentine formation, and regular preventive measures (e.g., topical fluoridation or desensitizing agents) may have already contributed to reducing hypersensitivity (Linner et al. 2021). This age-related factor may also help explain the higher hypersensitivity rate reported in the Oslo cohort. Moreover, the Norwegian study was confined to a single urban region and conducted in a clinical setting, whereas our investigation was carried out on a larger scale and outside dental clinics. Although clinics may offer improved diagnostic conditions, restricting the study to the Oslo area may limit the capture of potential urban–rural differences or other demographic variations. Moreover, in Germany, such an approach is less feasible because only a few individuals receive treatment in public clinics, unlike in Norway, where most children are integrated into the public dental service (Haque Afzal et al. 2023). It remains unclear to what extent these institutional and population-based differences contributed to the observed differences.

At the tooth level, the overall number of hypersensitive teeth in this study (Table 1) was also comparatively low, below the previously reported range of 10.7–34.7% in clinical investigations (Raposo et al. 2019; de Castro et al. 2021; Linner et al. 2021). However, methodological differences must be considered, as many prior publications did not use epidemiological designs or settings. In the descriptive findings of this study, teeth with post-eruptive enamel breakdown showed a higher frequency of hypersensitivity than those with only demarcated opacities (Table 2). Yet, the mixed-effects logistic regression model (Table 3) did not detect a statistically significant association between enamel breakdown and hypersensitivity (*p* = 0.693). This apparent discrepancy suggests that while breakdown may coincide with hypersensitivity more often in raw frequency data, the relationship may be masked by other confounders once multiple factors are accounted for in the model. Additionally, enamel breakdown alone does not guarantee increased sensitivity, since the size, depth (enamel vs. dentin), and presence of coexisting conditions (e.g., carious dentin) likely modulate how stimuli reach the pulp (Shiau 2012; Raposo et al. 2019). As we did not document defect size or depth in this study, our ability to evaluate their impact on hypersensitivity is limited. Future research involving more detailed lesion documentation could help clarify these findings further. However, contrary to previous studies (Raposo et al. 2019; Afzal et al. 2024), we did not observe a statistically significant association between caries diagnosis in first permanent molars and MIH-related hypersensitivity. This might be explained by the fact that in the present study, MIH and caries were assessed independently and a tooth was only documented as decayed or filled if the respective condition was clearly attributable to caries (Fresen et al. 2024). Furthermore, the overall low caries prevalence in this Bavarian cohort may have limited the statistical power to detect such an association.

Another noteworthy aspect of this investigation is the application of the MIH-TNI within an epidemiological cross-sectional study. While the MIH-TNI is increasingly used, particularly in studies examining the oral health-related quality of life in individuals with MIH (Bekes et al. 2021; Joshi et al. 2022; Sekundo et al. 2024), its deployment in large-scale epidemiological studies has not yet been reported.

In this cross-sectional examination, a significant proportion of children (49.7%) and teeth (26.2%) diagnosed with MIH presented an MIH-TNI score of 2, 3, or 4. In accordance with current EAPD severity grading, these findings are categorized as “severe” (Lygidakis et al. 2022). Nevertheless, there is substantial variability in reported figures for severe manifestations, ranging from 3.5% to 50.9% in Germany (Kühnisch et al. 2014; Amend et al. 2021), 46.8% in Norway (Afzal et al. 2024), and 23.5% to 31.7% globally (Schwendicke et al. 2018). It is important to interpret the classification of “severe” with caution, especially as no detailed information regarding defect size, extension, or dentin exposure was collected. It can be presumed that this large “severe” group within our study population is heterogeneous, ranging from small to more extensive defects of varied clinical importance. Consequently, any interpretation suggesting increasing severity in MIH patients should remain tentative. Interestingly, the majority of severe cases (MIH-TNI 2, 3, or 4) in this study fell into the MIH-TNI 2 category. Thus, hypersensitivity contributed less substantially to the overall classification of severity than did post-eruptive enamel breakdown or atypical restorations (Table 2).

This study has both strengths and limitations. A key strength is the chosen design, which involves a cross-sectional epidemiological examination that accounts for both caries and MIH in 5418 children, with a clinical assessment of MIH-related hypersensitivity rather than relying on questionnaires or asking children (Afzal et al. 2024). Furthermore, the application of the MIH-TNI in a large-scale epidemiological context is another notable strength. However, some limitations must be acknowledged. The relatively low individual participation rate of 50.4% restricts representativeness and, consequently, the generalizability of our findings. In addition, the agreement levels for intra- and inter-examiner reliability showed considerable variation (intra-rater: 0.484–1.000; inter-rater: 0.371–0.956), which may reflect differences in prior experience among the examiners as well as the interpretative variability inherent in image-based calibration. Although all values reached at least fair agreement according to international standards (Landis and Koch 1977), this heterogeneity should be acknowledged as a potential limitation, as it may affect the internal consistency of diagnostic outcomes. Another limitation concerns the assessment of hypersensitivity, which was recorded in a dichotomous manner (yes/no) and therefore did not allow for a quantitative assessment of pain intensity. Although the value of tools such as the Wong-Baker Faces Pain Rating Scale, visual analogue scale (VAS), or Schiff cold air sensitivity scale (SCASS) has been recognized (Gernhardt 2013; Idon et al. 2019), they were not employed due to their increased methodological requirements in this field trial. Moreover, the exact defect size and extent, as intended by the MIH-TNI, were not documented. Thus, the relationships between hypersensitivity and these variables cannot be analysed. Although defect size is highly relevant clinically, it faces a degree of subjectivity and is therefore also less feasible in large-scale epidemiological studies.

## Conclusion

Although MIH is relatively common, the overall prevalence of MIH-related hypersensitivity was low compared with that reported in similar studies. Descriptively, hypersensitivity appeared more frequent when enamel breakdowns were present. However, mixed-effects logistic regression did not confirm a statistically significant association between enamel breakdown and hypersensitivity. This suggests that potential confounders may obscure this relationship in a multivariate context. Further research is needed to clarify the differing hypersensitivity rates across studies, ideally through comparable epidemiological designs, and to explore additional contributing factors, such as the depth and extent of enamel breakdown.

## Data Availability

No datasets were generated or analysed during the current study.
